# Supplementary material for: Clinicopathological features and ethnic disparities of melanoma in the United Arab Emirates: 2017–2025
Source: PLoS One. 2026 Jun 5;21(6):e0340749. doi: 10.1371/journal.pone.0340749 (PMC13240860; doi:10.1371/journal.pone.0340749)
Supplement: S1 File — (PDF) [file pone.0340749.s001.pdf]

STROBE Statement—Checklist of items that should be included in reports of ***cross-sectional studies***

| | Item No | Recommendation |
|---|---|---|
| **Title and abstract** | 1 | (*a*) Indicate the study's design with a commonly used term in the title or the abstract |
| | | (*b*) Provide in the abstract an informative and balanced summary of what was done and what was found Pages 1-2, title and abstract |
| **Introduction** | | |
| Background/rationale | 2 | Explain the scientific background and rationale for the investigation being reported |
| Objectives | 3 | State specific objectives, including any prespecified hypotheses Page 3, lines 60-88, introduction |
| **Methods** | | |
| Study design | 4 | Present key elements of study design early in the paper Page 4, lines 93-103, methods 2.1 |
| Setting | 5 | Describe the setting, locations, and relevant dates, including periods of recruitment, exposure, follow-up, and data collection Page 4-5, lines 104-126, methods 2.2-2.3 |
| Participants | 6 | (*a*) Give the eligibility criteria, and the sources and methods of selection of participants Page 5, lines 127-136, methods 2.4 |
| Variables | 7 | Clearly define all outcomes, exposures, predictors, potential confounders, and effect modifiers. Give diagnostic criteria, if applicable Page 5, lines 137-150, methods 2.4 |
| Data sources/ measurement | 8* | For each variable of interest, give sources of data and details of methods of assessment (measurement). Describe comparability of assessment methods if there is more than one group Page 5, lines 137-150, methods 2.4 |
| Bias | 9 | Describe any efforts to address potential sources of bias Page 5, lines 151-159, methods 2.5 |
| Study size | 10 | Explain how the study size was arrived at N/A - Retrospective design |
| Quantitative variables | 11 | Explain how quantitative variables were handled in the analyses. If applicable, describe which groupings were chosen and why |
| Statistical methods | 12 | (*a*) Describe all statistical methods, including those used to control for confounding |
| | | (*b*) Describe any methods used to examine subgroups and interactions |
| | | (*c*) Explain how missing data were addressed |
| Page 6, lines 160-181, methods 2.6 | | (*d*) If applicable, describe analytical methods taking account of sampling strategy |
| | | (*e*) Describe any sensitivity analyses |
| **Results** | | |
| Participants | 13* | (a) Report numbers of individuals at each stage of study—eg numbers potentially eligible, examined for eligibility, confirmed eligible, included in the study, completing follow-up, and analysed Page 7-8, lines 189-221, results 3.1-3.2, figure 1 |
| | | (b) Give reasons for non-participation at each stage Page 7-8, lines 189-221, results 3.1-3.2, figure 1 |
| | | (c) Consider use of a flow diagram Figure 1 |
| Descriptive data | 14* | (a) Give characteristics of study participants (eg demographic, clinical, social) and information on exposures and potential confounders Table 1 |
| | | (b) Indicate number of participants with missing data for each variable of interest NA |
| Outcome data | 15* | Report numbers of outcome events or summary measures Page 8, lines 222-243, results 3.3, figures 2-4 |
| Main results Page 8-10, lines 222-270, results 3.3 and 3.5, figures 2-4, and table 2 | 16 | (*a*) Give unadjusted estimates and, if applicable, confounder-adjusted estimates and their precision (eg, 95% confidence interval). Make clear which confounders were adjusted for and why they were included |
| | | (*b*) Report category boundaries when continuous variables were categorized |
| | | (*c*) If relevant, consider translating estimates of relative risk into absolute risk for a meaningful time period |
| Other analyses | 17 | Report other analyses done—eg analyses of subgroups and interactions, and sensitivity analyses Page 10, lines 245-256, results 3.5, figure 5 |

| | | |
|---|---|---|
| **Discussion** | | |
| Key results | 18 | Summarise key results with reference to study objectives Page 12, lines 293-299 |
| Limitations Page 16, lines 433-442, Limitations | 19 | Discuss limitations of the study, taking into account sources of potential bias or imprecision. Discuss both direction and magnitude of any potential bias |
| Interpretation Pages 12-14, lines 300-392 | 20 | Give a cautious overall interpretation of results considering objectives, limitations, multiplicity of analyses, results from similar studies, and other relevant evidence |
| Generalisability | 21 | Discuss the generalisability (external validity) of the study results Pages 15-16, lines 393-431 |
| **Other information** | | |
| Funding | 22 | Give the source of funding and the role of the funders for the present study and, if applicable, for the original study on which the present article is based N/A |

*Give information separately for exposed and unexposed groups.

**Note:** An Explanation and Elaboration article discusses each checklist item and gives methodological background and published examples of transparent reporting. The STROBE checklist is best used in conjunction with this article (freely available on the Web sites of PLoS Medicine at http://www.plosmedicine.org/, Annals of Internal Medicine at http://www.annals.org/, and Epidemiology at http://www.epidem.com/). Information on the STROBE Initiative is available at www.strobe-statement.org.
